# Bacterial Stressors in Minimally Processed Food

**DOI:** 10.3390/ijms10073076

**Published:** 2009-07-08

**Authors:** Vittorio Capozzi, Daniela Fiocco, Maria Luisa Amodio, Anna Gallone, Giuseppe Spano

**Affiliations:** 1 Department of Food Science, University of Foggia, via Napoli 25, 71100 Foggia, Italy; E-Mail: vittorio.capozzi@gmail.com (V.C.); 2 Department of Biomedical Sciences, University of Foggia, via L. Pinto 1, 71100 Foggia, Italy; E-Mails: d.fiocco@unifg.it (D.F.); a.gallone@unifg.it (A.G.); 3 Department of Production Sciences, Engineering, and Economics for Agricultural Systems (PrIME), University of Foggia, via Napoli 25, 71100 Foggia, Italy; E-Mail: m.amodio@unifg.it (M.L.A.)

**Keywords:** stress, stressors, fresh cut, pathogens

## Abstract

Stress responses are of particular importance to microorganisms, because their habitats are subjected to continual changes in temperature, osmotic pressure, and nutrients availability. Stressors (and stress factors), may be of chemical, physical, or biological nature. While stress to microorganisms is frequently caused by the surrounding environment, the growth of microbial cells on its own may also result in induction of some kinds of stress such as starvation and acidity. During production of fresh-cut produce, cumulative mild processing steps are employed, to control the growth of microorganisms. Pathogens on plant surfaces are already stressed and stress may be increased during the multiple mild processing steps, potentially leading to very hardy bacteria geared towards enhanced survival. Cross-protection can occur because the overlapping stress responses enable bacteria exposed to one stress to become resistant to another stress. A number of stresses have been shown to induce cross protection, including heat, cold, acid and osmotic stress. Among other factors, adaptation to heat stress appears to provide bacterial cells with more pronounced cross protection against several other stresses. Understanding how pathogens sense and respond to mild stresses is essential in order to design safe and effective minimal processing regimes.

## 1. Introduction

**Figure 1.**
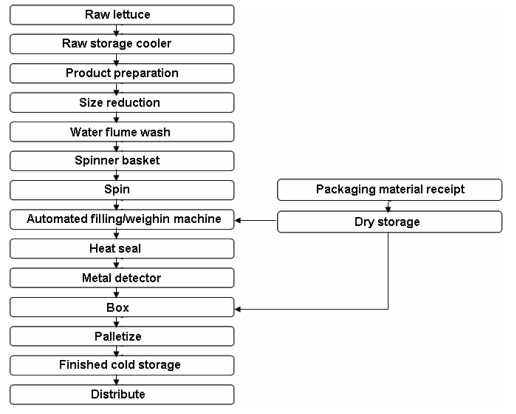
Example of fresh-cut lettuce operation.

Different organizations (WHO, FAO, USDA, EFSA) recommend the regular consumption of fruit and vegetables for promoting/maintaining good health. Freshly prepared, ready-to-eat fruits and vegetables are a good example of convenient foods within the context of our modern life. Driven by consumers’ tendencies, the fresh-cut fruit and vegetable industry has expanded rapidly in recent years; as a consequence the production and manufacture of these products is at a stage of innovative dynamics. Consumers require high quality and convenience; to harmonize these demands without compromising safety, it is necessary to implement new preservation technologies. Moreover, some of these new preservation technologies aim at energy saving and being environmentally friendly. Fresh-cut are raw fruits and vegetables that have been washed, peeled, sliced, chopped or shredded prior to being packaged for consumption. These products are typically preserved within semi-permeable packages and stored at refrigeration temperatures. The unit operations in use usually led to the destruction of surface cells making available a potentially richer source of nutrients for microorganisms [1,2]. These factors combined with high a_w_ and either close to neutral (vegetables) or low acidic (many fruits) tissue pH, make easy rapid microbial growth [3,4]. A number of important human pathogens can contaminate fresh-cut produce and there has been an augment in the number of food produce-linked foodborne outbreaks in recent years [5]. In food processing, “mild technologies” are used to describe the technologies for the storage or processing of foods that, in principle, allow to minimize the thermal damage, mechanical and oxidative and chemical and biological contamination that usually accompany such operations unit. Many of these mild preservation technologies aim at being energy saving and environmentally friendly. Ohlsson [6] suggested that minimal processing techniques have emerged to replace traditional harsher methods of food preservation as they retain nutritional and sensory quality better. “Minimal processing” describes non-thermal technologies to process food in a manner to guarantee the food safety and preservation as well as to preserve as much as possible the fresh-like attributes of fruits and vegetables (Figure 1).

This has stimulated interest in the use of mild preservation procedures and the development of the combined effects of several antimicrobial principles in multifactorial preservation approach or hurdle technology [7]. This approach is based on the observation that antimicrobial factors act co-operatively or synergistically with their combined antimicrobial effect being greater than the sum of the individual factors. Numerous reports described such observations for a range of different hurdles [8,9]. The practical outcome of this is that the combined effect of several relatively mild antimicrobial hurdles may offer the desired shelf life and safety properties while retaining many desirable sensory characteristics.

Targeted application of the hurdle concept has become more available as a result of the important improvements in our understanding of the principles of main preservative factors and their interactions [10]. On exposure to stressful conditions such as drying, cold, heat and low pH, stressed bacterial cells may lose their viability, become injured, or express adaptive mechanisms that would help them to survive or even continue growth during stress. These mechanisms begin with stress-sensing followed by producing signals that induce the development of a response that aids adaptation. On sensing stress and developing a signal, cells synthesize mechanisms to cope with the emergent hardship. These mechanisms involve modifications of gene expression and protein activities aiming at preventing or reducing injures to cellular structures and components.

In this paper, we review the molecular basis of bacterial stress response to classical and new stressors that deal with some mild technologies and minimal processing. Our goal is to stimulate the research and the development of molecular targets in order to analyse bacterial cell response in minimally processed food and, particularly, “cross protection”. This biological mechanism, might have an important role in optimizing food preservation procedures and improve process sustainability and global quality of fresh-cut produce, from safety to the healthy properties.

## 2. Bacterial Pathogens

Food-produce contamination can occur during agricultural production (via animals or insects, soil, water, dirty equipment and human handling), harvesting, processing (cutting, shredding, washing, contaminated work surfaces/equipment, hygiene practices of workers), packaging (contaminated packaging materials/equipment) and transportation and distribution.

*Salmonella* is the most common cause of disease outbreaks linked to fresh fruit and vegetables. Salmonellae are abundant in faecal material and sewage-polluted water; consequently they may contaminate soil and crops with which they come into contact. Salmonellae from a range of food-produce, including sprouted seeds, cantaloupe melons, tomatoes, unpasteurised citrus juices, rocket and lettuce, have been responsible for several food poisoning outbreaks [11].

*Escherichia coli* O157:H7 is a member of the enterohemorrhagic group of pathogenic *E. coli* that has emerged as a foodborne and waterborne pathogen of major public health concern [12]. Fresh produce was not considered a significant vector for the transmission of *E. coli* O157:H7 until the mid-1990s, when a series of outbreaks associated with minimally processed horticultural products clearly showed that contamination can occur by indirect routes [13,14]. The largest *E. coli* O157:H7 outbreak ocurred in 1996, when >6,000 school children in Japan were infected with *E. coli* O157:H7 from white radish seed sprouts. Since 1993, 26 reported outbreaks of *E. coli* O157:H7 infection have been traced to contaminated lettuce and leafy green vegetables [15]. A recent multi-state outbreak in the USA linked to bagged fresh spinaches caused approximately 205 confirmed illnesses, 31 cases of hemolytic uremic syndrome and three deaths [16,17]. This outbreak was followed by two restaurant associated outbreaks linked to the consumption of pre-washed lettuce [18]. These recent outbreaks have highlighted the dangers of centralised distribution and the great distances that fresh produce travels.

The Gram-positive bacterium *Listeria monocytogenes* is a food-borne pathogen of both public health and food safety significance. *L. monocytogenes* is widespread on plants and on agricultural environment generally, and is capable of surviving a variety of environmental stresses, including refrigeration temperatures [19], gas atmospheres commonly present within modified atmosphere packaging (MAP) produce and relatively low pH. Although there is a large amount of literature dedicated to researches concerning with the isolation, attachment, survival and growth of *L. monocytogenes* on food-produce [20,21], only two fresh-cut produce related listeriosis outbreaks have been documented [22,23].

## 3. Key Factors Affecting the Survival and Growth of Pathogens

Pathogen survival and growth on food-produce is influenced by a number of interdependent factors, principally storage temperature, product type/combinations, minimal processing operations (e.g. slicing, shredding, washing and decontamination treatments), mild technologies, package atmosphere and competition from the natural microflora present on food-produce. First, each product type has an exclusive combination of compositional and physical characteristics and will have specific growing, harvesting and processing practices, and storage conditions; pathogen growth on food-produce varies significantly with the type of product [24,25]. Storage of food-produce at adequate refrigeration temperatures is probably the single most important factor affecting survival and growth of pathogens. Washing with tap water removes soil and other debris, some of the surface microflora, cell contents and nutrients released throughout slicing that support growth of microorganisms [26]. However, while washing in tap water removes bacteria from exposed surfaces, substantial numbers will remain in hollows at the connection of epidermal cells and in folds in the epidermis [27–29]. In addition, due to the re-use of wash water in industry, washing may result in bacterial enrichment and cross-contamination of products rather than decontamination [3,30]. A variety of antimicrobial wash solutions have been used to diminish populations of microorganisms on fresh produce. Chlorine added to water as a solid, liquid or gas is the most frequently used disinfectant for fresh fruits and vegetables [30,31]; however, a wide variety of other disinfectants, including acidic electrolysed water [32], peroxyacetic acid [33], chlorine dioxide [34], hydrogen peroxide [35], organic acids [36], trisodium phosphate [34] and ozone [37] have been also evaluated [38]. Natural antimicrobials from edible plants such as oilseeds, herbs, spices, fruit and vegetables, have been studied for their potential as possible replacements for chemical additives because of their safety for human consumption and broad acceptance from consumers [39,40]. Phenolic compounds present in plant essential oils (EOs) have been shown to possess antimicrobial activity and some are classified as generally recognised as safe (GRAS), and consequently may be useful to prevent post-harvest growth of spoilage and pathogenic bacteria [41]. Efforts to improve the overall effectiveness of the washing step by the use of classical physical treatments, such as mild heat or ethanol vapours, may enhance pathogen destruction. Novel decontamination techniques, including ultra-violet (UV) irradiation, high-pressure treatment, pulsed electric fields, microwave, high intensity pulsed light and thermal destruction using condensing steam, warrant supplementary investigation [42]. When a fresh-cut product is packaged, it continues to breath thereby modifying the gas atmosphere inside the package, hence the term modified atmosphere packaging (MAP). Ideally, O_2_ levels will fall from 21% in air to 2–5%, and CO_2_ levels will increase to the 3–10% range. The gas mixtures control the product’s biochemical and enzymatic reactions, inhibit the growth of microorganisms and make longer the shelf-life.

MAP produce harbour large populations of microorganisms including pseudomonads, lactic acid bacteria (LAB) and Enterobacteriaceae [28,43]. The background microflora supply indicators of temperature abuse largely by causing detectable spoilage, and levels can vary considerably for each product and during storage. LAB can exert antibacterial effects due to one or more of the following mechanisms: lowering the pH; generating H_2_O_2_ [44]; competing for nutrients; and by producing antimicrobial compounds, such as bacteriocins [45]. The bacteriocins alone were also used in biocontrol of fresh-cut microflora. One other biotic factor to control bacterial pathogens on fresh-cut produce is represented by the use of bacteriophages.

## 4. Stressors and Related Bacterial Stress Response

**Figure 2.**
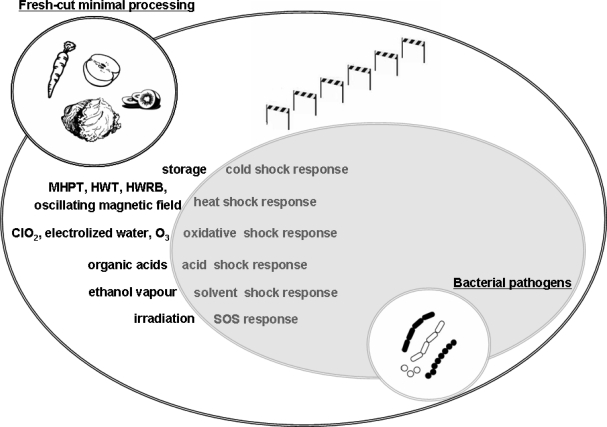
Exemplificative interdependent factors that influence pathogens survival and growth on fresh-cut minimal processing [mild heat pre-treatments (MHPT); hot water immersion treatment (HWT); hot water rinsing and brushing (HWRB); oscillating magnetic fields (ohmic heating, dielectric heating, microwaves)]. Combined effects of several antimicrobial strategies is known as “hurdle technology”.

Acid adapted *L. monocytogenes*, *Salmonella* and *E. coli* O157:H7 were shown to survive significantly better in acidic foods such as fruit juices, than their non-acid adapted counterpart cells [46,47]. This is due to a specific stress response characteristic of acid stressor, that involved the induction of a specific subset of genes organized into regulons, constituting the acid shock stimulon. A general overview of the stress responses to each single key factor affecting pathogen survival, is shown in Figure 2.

### 4.1. Cold Stress

Exposure to cold temperatures following harvest in order to minimize and/or inhibit the effects of wounding stress is recognized as one of the principal factors controlling the quality of fresh-cut leafy vegetables [48,49]. Although 0 °C is usually the desirable temperature for most fresh-cut products, in practice many of them are shipped and marketed at temperatures ranging from 5 to 10 °C [50].

In response to temperature downshift, a number of changes take place in prokaryotic cellular physiology such as, (i) decrease in membrane fluidity, (ii) stabilization of secondary structures of nucleic acids leading to reduced efficiency of mRNA translation and transcription, (iii) inefficient folding of some proteins, and (iv) hampered ribosome function [51]. A number of cold shock proteins are induced to cope with these harmful effects of temperature downshift. For all organisms, maintenance of functional cell membranes is a limiting factor for survival. Upon cold shock the physical status of biological membranes is altered from being fluid to becoming rigid. In a process generally termed homeoviscous adaptation, with decreasing temperature, bacteria incorporate fatty acids of lower melting points into lipids in a species-specific mode to re-establish membrane integrity and hence function. In this picture, the introduction of double bonds into acyl chains can either be achieved anaerobically during fatty acids synthesis or aerobically by modification of readily synthesized fatty acids through fatty acid desaturase enzymes. Transcription and translation are closely coupled in bacterial cells. However, transcription machinery and ribosomes generally occupy different subcellular regions in bacteria such as *Escherichia coli* and *Bacillus subtilis*, indicating the need for (a) mechanism(s) coupling these processes. A prime function of this mechanism(s) would be ensuring the transfer of unfolded mRNA from the nucleoid to the ribosomes, which need linear mRNA for the initiation of translation. During conditions of a sudden decrease in temperature (cold shock), secondary structures in mRNA would pose an even greater problem for the initiation process. Two conserved classes of proteins, cold shock proteins (CSPs) and cold induced RNA helicases (CSHs), appear to be key players in the prevention of secondary mRNA structures and in transcription/translation coupling. CSPs are general mRNA-binding proteins, and like CSH-type RNA helicases, the presence of at least one csp gene in the cell is essential for viability [52]. *E. coli* contains nine CSPs (CspA to CspI), of which four (CspA, -B, -G, and -I) are cold shock inducible [53]. CspA, CspC, and CspE are RNA-binding proteins which function as transcriptional antiterminators by preventing the formation of secondary structures in the nascent RNA. Csp-induced transcriptional antitermination is responsible for the increased expression of several genes [54,55]. *B. subtilis* contains three CSPs (CspB, -C, and -D); CspB is essential for cellular growth in a strain lacking CspC and CspD and plays an important role for efficient protein synthesis at optimal and low temperatures [56], while CspB and CspC are major stationary phase-induced proteins [57].

### 4.2. Heat Stress

The use of hot water or steam may be a possibility to replace disinfection. Martín-Diana *et al.* [58] reported that short time exposure of fresh-cut (FC) lettuce to water steam reduced the respiration rate (RR), partially inactivated browning-related enzymes, and kept the mesophilic load as low as with a chlorine treatment. In FC fruits, mild heat pre-treatments (MHPT) (40 °C/70 min or 46 °C for 75 min) were effective in inducing firmness and avoiding browning of the cut surface while preserving their nutritional quality [59,60]. In the post-harvesting of fresh-cut vegetables hot water immersion treatment (HWT) and hot water rinsing and brushing (HWRB) technologies were successfully used [61]. HWT is applied at temperatures between 43 °C and 53 °C for periods of several minutes for the fresh cut, while HWRB is employed commercially for 10-25 s at temperatures between 48 °C and 63 °C. Additionally, oscillating magnetic fields (ohmic heating, dielectric heating, microwaves) represent alternative ways to heating food matrices. The application of high temperatures has been widely used for the elimination of foodborne pathogens. This is due to the effectiveness of heat and its ability to cause damage to diverse structures and components in microbial cells including outer and cytoplasmic membranes, RNA and DNA. It also causes protein denaturation leading to destruction of enzyme activity and enzyme-controlled metabolism in microorganisms [62,63]. Traditional heat treatment techniques widely adopted in food industry, such as pasteurization and sterilization are too harsh to be resisted by vegetative bacterial cells. An interesting concept has been also proposed on sensing environmental stresses including heat. According to this theory, cells produce extracellular proteins to sense stress and act as “alarmones” of the emergent environmental changes [64]. The production of these extracellular components are suggested to provide early warning of stress compared to the cytoplasmic membrane and ribosomes. Following stress-sensing, cells set up strategies to cope with the emergent hardship. These mechanisms involve changes of gene expression and protein activities aiming at preventing or reducing damage to cellular structures and components. An important change is the induction of the synthesis of the so-called “heat shock proteins” (HSPs) [65]. These are highly conserved proteins that act as molecular chaperones or proteases affecting protein folding, repair and degradation under normal and stress conditions [66]. For example, during heat stress, several HSPs such as DnaJ, DnaK, GrpE and GroEL function as chaperones preventing or repairing protein misfolding and thus ensuring their proper functioning. Whereas, other HSPs including ClpP, ClpX and Lon act as proteases catalyzing the degradation of misfolded proteins generated by exposure to stress. Both functions of HSPs help to provide cells with functional proteins which allow survival or growth during heat stress. In a study on heat shock proteins synthesis and heat resistance of S*almonella typhimurium*, Mackey and Derrick [67] found four important heat shock proteins, with molecular weights ranging from 25 to 83 KDa, and correlated them with DnaK and GroEL reported in *E. coli*. The chaperone GroEL was found to diminish heat inactivation of a range of enzymes *in vitro* [68].

### 4.3. Acid and Solvent Stress

A decontamination step with lactic acid was evaluated to reduce the microbial contamination of minimally processed vegetables [69]. The antimicrobial effect of 1% and 2% lactic acid was established in potable tap water. Citric acid has been widely accepted as effective in reducing superficial pH of cut fruits [70]. Well documented is the antimicrobial effect of the treatments based on calcium salts on fruits and vegetables [58]. While calcium has a prevalent technological importance, on the other hand, organic calcium salts, with lowering pH (i.e. calcium lactate), may have antimicrobial properties [71].

In recent years, the acid stress response of several prokaryotes has been studied with both proteomics and transcriptomics approaches. A few reports describe the use of these approaches to study the organic acid stress response caused by lactate [73], acetate [74], propionate [74] and formate [75]. In other prokaryotes some enzymatic-transport systems have been found to play a role in pH control or in the maintenance of the proton motive force: a proton-translocating F_1_ F_0_ -ATPase [76]; several sodium-proton antiporters [77]; amino acid decarboxylases that use an intracellular hydrogen ion for the decarboxylation of an imported amino acid [76,78]. Several theories have been postulated to explain the toxic effect of organic acids in more detail. One of these considers organic acids as uncouplers that transport protons towards the inside of the cell, which is a pH-driven process. Eventually, this influx could lead to a complete dissipation of the proton motive force [79]. A second aspect relates to the deleterious effects of the lower intracellular pH, caused by the lactic acid. However, whereas many organisms aim at maintaining a constant intracellular pH [80], most anaerobic fermenting species avoid a basic pH keeping a lower intracellular pH, and, as a result, increasing their tolerance to organic acids [81]. A third factor explaining the inhibitory effect of organic acids is the intracellular accumulation of anions, which could lead to both end-product inhibition and a loss of water activity (a_w_) [82].

A link between acid, ethanol and stress response has been demonstrated in a number of Gram-positive bacteria including food-borne pathogens. The expression of some stress inducible genes identified so far in bacteria is also affected by low pH values and ethanol, suggesting the intersection of different regulatory pathways and overlapping control of gene expression. For instance, acid and solvents such as ethanol and buthanol, induces several heat shock proteins, including DnaK and GroEL. Moreover, the Clp-ATP dependent proteases which degrade aberrant and nonfunctional proteins, arising from stress conditions are also responsible for adaptation to multiple stresses and are inducible by low pH or high ethanol [83].

Ethanol kills organisms by denaturing their proteins and dissolving their lipids and is effective against most bacteria, but is ineffective against bacterial spores [84]. Additionally, ethanol causes water stress by lowering a_w_ and thereby interferes with hydrogen bonds within and between hydrated cell components, ultimately disrupting enzyme and membrane structure and function [85].

Plotto *et al.* [86] tested ethanol vapours on fresh cut fruit and showed that at lower application rates (8–10 h exposure), ethanol could be used as a safe microbial control in a fresh-cut production sanitation system. Ethanol vapors applied to whole apples reduced ethylene and CO_2_ production of fresh-cut apples, and their shelf life was increased due to maintenance of visual quality [87].

### 4.4. Oxidative Stress

Chlorine dioxide is a stable dissolved gas, having a high oxidation and penetration power; ClO_2_ is a strong bactericide: with minimal contact time, it is highly efficient against pathogenic organisms such as *Legionella*, *Amoebal cysts*, *Giardia cysts*, *E. coli*, and *Cryptosporidium* [88]. Hyrdogen peroxide is a powerful bactericide (including spores) and oxidant, being able to generate other cytotoxic oxidising chemical species such as hydroxyl radicals [89]. Electrolyzed water (EW) is formed by adding a very small amount of NaCl (usually about 0.1%) to pure water, and conducting a current across an anode and cathode [90], the cathode area produces alkaline reducing water while the anode area produces acidic oxidizing water [90]; upon release of O•, O_3_ acts as a strong oxidizing agent being very effective in destroying microorganisms [91]. O_3_ destroys microorganisms by the progressive oxidation of vital cell components, preventing microbial growth and extending the shelf-life of many fruit and vegetables, and its industrial use is increasing [92].

Oxidative stress is a key stress in bacteria, caused by an imbalance between intracellular oxidant concentration, cellular antioxidant protection and oxidative change of macromolecules (membrane lipids, proteins and DNA repair enzymes) [93,94]. The reactive oxygen species (ROS) and nitrogen species (RNS) are the main causes of oxidative stress [95]. They are principally constituted by the hydroxyl radical (^•^OH), the superoxide anion (O_2_^−^), hydrogen peroxide (H_2_O_2_), organic hydroperoxide (ROOH), peroxynitrite (OONO) and nitric oxide (NO). ROS and RNS cause damages to proteins [96], DNA molecules [97], RNA and lipids leading to negative repercussions of the cellular metabolism functions [98]. The toxicity of ROS/RNS discloses the significant role of competent protection subsystems, for instance the detoxification subsystem that numbers enzymes classified with regard to their substrates, or thioredoxin that help in the cellular defense against several oxidative stresses [99]. Catalases are common enzymes found in almost all-living organisms that catalyze the decomposition of hydrogen peroxide to produce oxygen and water [100]. Peroxidases reduce hydrogen or organic peroxides into water and alcohol moiety. This class of enzymes includes a wide number of phylogenetically unrelated families such as peroxiredoxins [101], rubrerythrins [102], glutathione-peroxidases [103] or haloperoxidases [104]. Superoxide dismutases (SOD) dismute superoxide into hydrogen peroxide and oxygen [105]. An additional mechanism lately reported involves superoxide reductases (SOR), that are non-heme iron proteins [106]. The latter catalyzes the one-electron reduction of superoxide into hydrogen peroxide. Moreover, RNS-scavenging enzymes are essentially globins and nitric oxide reductases [107].

### 4.5. Osmotic Stress

The inner osmotic pressure of a bacterial cell is normally maintained higher than that of the surrounding medium [108,109], and this is generally referred to as “turgor pressure”. To maintain this turgor pressure in a medium with a high concentration of solutes and to mitigate the osmotic stress resulting from low water availability, microbial cells tend to increase their internal cytoplasmic solute concentration through different mechanisms [110]. Water availability in a food environment is usually assessed by measuring the water activity (a_w_). Water activity (a_w_) is defined as the ratio of the water vapour pressure of the food or solution to that of pure water at the same temperature. It indicates the quantity of water available in a material for microbial growth. The values of water activity are represented in a scale range between 0 (no available water) and 1 (pure water). While most bacteria show rapid growth at high a_w_ (0.99), microbial growth does not occur at a_w_ below 0.6 [111]. Several osmosensors are known to be involved in osmotic stress induced responses [112]. As mentioned above, the solute concentration in the bacterial cytoplasm is normally maintained above that of the external environment. However, immediately, following an osmotic up-shift (decrease in a_w_) in the environment, bacterial cells respond by activation of transporters that aid the cell increase the internal solute concentration by either uptake of inorganic ions into the cell or synthesis and concentration of specific organic solutes to counter the osmotic stress [113]. Under mild osmotic stress, only the ionic solutes are accumulated, whereas other compatible solutes become progressively more important on exposure to severe osmotic stress [114]. These accumulated solutes must not interfere with biochemical processes within the cell and they are thus termed “compatible” solutes [108]. Potassium ions (K^+^), glutamate (as ionic solutes), glycine betaine, trehalose and proline (as non ionic solutes) are the most important compatible solutes accumulated by bacterial cells [114,115]. Members of the *Enterobactereaceae* family are reported to synthesize glutamate and trehalose, while K^+^, glycine betaine and proline are taken up from the medium [110]. Accumulation of K^+^ during osmotic stress takes place in the initial response, which is then accompanied by increased synthesis of glutamate to preserve electroneutrality in the cytoplasm. This is followed by the accumulation of other compatible solutes such as glycine betaine, proline and trehalose [114,115]. The accumulation of the latter molecules was found to influence that of the originally accumulated compatible solutes since increased levels of trehalose were associated with decreased accumulation of K^+^ and glutamate [114]. The above mechanism of solute accumulation appears to be affected by environmental temperatures as trehalose accumulation was reported to be enhanced at higher temperatures (45°C) in *S. typhimurium* [116].

### 4.6. Irradiation

Radiations have been used both to delay ripening-associated processes and to diminish microorganism growth. Several studies have been published, and in recent times, UV-C has been used as an alternative treatment to preserve the quality of different fruits and vegetables [117]. The use of non-ionizing, germicidal and artificial UV at a wavelength of 190–280nm (UV-C) was found to be effective for surface decontamination of fresh-cut products. Lado and Yousef [118] reported that UV-C radiation from 0.5 to 20 kJm^−2^ inhibited microbial growth by inducing the formation of pyrimidine dimers which alter the DNA helix and block microbial cell replication. Therefore, cells which are unable to repair radiation damaged DNA die and sub-lethally injured cells are often subject to mutations. Treatment with ultraviolet light is simple to use and lethal to most types of microorganisms [119]. Intense light pulses (ILP) are an interesting decontamination method for food surfaces approved by the US Food and Drug Administration (FDA) that could be appropriate for disinfecting fresh-cut produce. ILP kills microorganisms using short time (from 85 ns to 0.3 ms) high frequency pulses (from 0.45 to 15 Hz) and energy per pulse ranging from 3 to 551 J of an intense broad spectrum, rich in UV-C light [120]. This treatment seems to induce structural changes of microbial DNA, similarly to the effect caused by continuous UV sources, although further mechanisms seem to be involved [121].

Irradiation of DNA with UV light produces a variety of photoproducts, of which the main species are cyclobutane pyrimidine dimers (CPDs) and pyrimidine-pyrimidone. Both lesions, if not repaired, provoke mutagenesis and cell death. To survive in a UV-rich environment, *E. coli* developed an inducible response known as the SOS response regulated by the *recA-lexA* regulon. The SOS response aids survival by combining increased expression of genes involved in Nucleotide Excision Repair (NER) and recombinational repair mechanisms. The genes *recA* for the recombination enzyme, RecA, and *uvrA* and *uvrB* for subunits of the UvrABC NER enzymes, UvrA and UvrB, have SOS boxes that are bound by the LexA repressor under physiological conditions. Upon UV irradiation, the constitutive amount of RecA protein binds single-stranded DNA resulting from replication blocks and acts as a coprotease for inactivation of LexA, consequently the levels of RecA, UvrA and UvrB increase. Upon completion of repair, the inducing signal disappears and cells return to the preinduction state [122].

### 4.7. High Pressure Stress

High pressure treatments of fruits and vegetables have been applied on processed products typically having been processed to some degree [123,124]. High pressures have been used to inhibit enzymes, microorganisms and spores, and to preserve aroma compounds [125]. Yanga *et al.* [126] indicate an undesirable effect of hyperbaric storage on the synthesis of peach volatiles immediately after storage, however, the post-storage potential for recovery of normal synthesis has not been assessed.

Pressure effects on any physiological or biochemical system basically result from the compression of the system, according to Le Chatelier’s principle, which states that at equilibrium a system tends to minimise the effects of troubling external factors. In lipid membranes, a pressure increase of 1,000 atm is equivalent to a temperature decrease of 20 °C [127]. Pressure increase and temperature decrease result in similar effects, i.e. by ordering structures and reducing flexibility in lipids, nucleic acids and carbohydrates [128]. For proteins, pressure and temperature act in synergy and promote protein denaturation and loss of function [129]. In *E.coli* O 157:H7, high pressure affected the transcription of many genes involved in a variety of intracellular mechanisms, including the stress response, the thioldisulfide redox system and the Fe-S cluster assembly [130].

### 4.8. Modified Atmosphere Packaging (MAP)

Producers mainly rely on produce sanitation, refrigeration temperatures, and, more recently MAP to extend shelf life and to reduce microbial load [131]. Modifying the internal atmosphere of a package lowers the oxygen (O_2_) concentration, from 20% to 0%, hence slowing down the growth of aerobic organisms and the speed of oxidation reactions. The removed oxygen can be replaced with nitrogen (N_2_), commonly acknowledged as an inert gas, or carbon dioxide (CO_2_), which can inhibit the growth of bacteria. Although there is a wide literature describing how MAP affects microbial load on various food-produce [132,133], little is known about how growth under subatmospheric oxygen partial pressures would impact the enteric pathogens’ ability to breach the gastric stomach barrier and increase the risk of disease. CO_2_ inhibits the growth of bacteria by (i) affecting cellular enzymes and decreasing the rate of metabolic reactions, (ii) CO_2_ product repression of carboxylases and decarboxylases, (iii) disrupting cell membrane structural integrity and/or specific functions, (iv) decreasing the substrate and intra-cellular pH, or by a combination of these mechanisms [134]. The extent of inhibition by CO_2_ varies with the microorganism, CO_2_ concentration, temperature of incubation, and type of food [134,135].

### 4.9. Biological Compounds

Greater consumer awareness and concern regarding synthetic chemical additives have led researchers and food processors to look for natural food additives with a broad spectrum of antimicrobial activity [136]. This is an heterogeneous category: some examples. Plant essential oils and natural aroma compounds are gaining interest for their potential as preservative ingredients or decontaminating treatments, as they have GRAS status and a wide acceptance from consumers [137,138]. The antimicrobial components are commonly found in the essential oil fractions and it is well established that many have a wide spectrum of antimicrobial activity, with potential for control of *L. monocytogenes* and spoilage bacteria within food systems [139]. Oregano (*Origanum vulgare*) and thyme (*Thymus vulgaris*) are amongst the most active EOs, while lemon balm (*Melissa officinalis*) and marjoram (*Origanum majorana*) exhibit a good antimicrobial action against Gram-positive and Gram-negative bacteria, respectively [140]. Chitosan, which is a cationic polysaccharide extracted from source of shellfish exoskeletons or the cell walls of some microorganisms and fungi, has been used to preserve the quality of post-harvest fruits and vegetables [141,142]. Martin-Diana *et al.* [143] tested whey permeate at different concentrations (0.5%, 1.5% and 3%) in the washing treatment of lettuce and carrots, and the results suggest that whey permeate could be a promising alternative for sanitizing fresh-cut vegetables.

### 4.10. Antagonistic Microflora

Packaged produce harbour large populations of microorganisms including pseudomonads, lactic and bacteria (LAB) and *Enterobacteriaceae* [28,43]. The background microflora provide indicators of temperature abuse largely by causing detectable spoilage, and levels can vary appreciably for each product and during storage. LAB can exert antibacterial effects due to one or more of the following mechanisms: lowering the pH; generating H_2_O_2_; competing for nutrients; and by producing antimicrobial compounds, such as bacteriocins [45]. Cai *et al.* [144] reported that a large portion of LAB isolates from beansprouts inhibited the growth of *L. monocytogenes*. Strains of LAB were reported to inhibit *Aeromonas hydrophila*, *L. monocytogenes* and *S. typhimurium*, on vegetable salads [145]. Various researchers have reported antagonism by the native microflora of vegetables against *Listeria* [146,147]. Reducing the background microflora of endive leaves and shredded lettuce resulted in enhanced growth of *Listeria* [148]. However, the inhibitory effects were dependent on gas atmosphere; in 3% O_2_ (balance N_2_) growth of the mixed population was inhibited while *L. monocytogenes* proliferated [149]. *Enterobacter* isolates significantly reduced *L. monocytogenes* growth during storage on a model lettuce medium; however, the inhibitory activities of *Enterobacter* decreased as the concentration of CO*_2_* increased [149]. Competitive microflora had a significant effect on the growth of *E. coli* O157:H7 in broth media [150]. Little is known about the mechanism by which *Salmonella* manages to compete with natural microflora and survive on plant products [151]. Generally, the complex interactions with the indigenous microflora may have significant effects on survival, growth and biocontrol of pathogens.

### 4.11. Bacteriocins

Bacteriocins are antimicrobial peptides or proteins produced by strains of different bacterial species. The antimicrobial activity of this set of natural substances against foodborne pathogenic, as well as spoilage bacteria, has raised considerable interest for their application in food preservation [152].

Nisin is the only commercially available bacteriocin recognized as a safe and legal biological food preservative (number E234) by the Food and Agriculture Organization and World Health Organization as well as the FDA. Nisin, a broad-spectrum, pore-forming bacteriocin, is produced by lactic acid bacteria that are often found on food-produce [153]. It is active against many Gram-positive bacteria, including *L. monocytogenes* [154]. Nisin is particularly active at the lower pH values typical of many fruits and some vegetables [155].

The production of bacteriocin must thus be coupled with a mechanism by which the producing strain can protect itself from the lethal action of its own antimicrobial compound. This mechanism is referred to as immunity. In non-nisin-producing *Lactococcus lactis*, nisin resistance could be conferred by a specific nisin resistance gene (*nsr*), which encodes a 35-kDa nisin resistance protein (NSR). NSR is a nisin-degrading protease [156], however, the mechanism underlying NSR-mediated nisin resistance is poorly understood.

### 4.12. Bacteriophages

Leverentz *et al.* [157] reported a study on the control of *Salmonella* by phages on fresh-cut fruits. The use of naturally occurring lytic phages to reduce contamination of fresh-cut produce with foodborne pathogens has several advantages over the use of chemical sanitizers and washes [38]. For example, methods commonly used in industry, such as aqueous washes containing chlorine formulations or plain water, are nonspecific and can achieve a less-than-10-fold reduction in *Listeria* populations on cut-produce surfaces [158]. Conversely, specific phages attack the targeted pathogens only, thus preserving the competitive potential of the indigenous microflora [38]. Leverentz *et al.* [159] found that treatment with a *Listeria*-specific lytic phage cocktail alone or in combination with nisin is an effective method for reducing *L. monocytogenes* contamination on fresh-cut fruit.

Bacteria have evolved different sophisticated natural bacteriophage defense systems that can interfere with bacteriophage proliferation at different steps during the lytic cycle. These consist of natural phage defense mechanisms that impede the adsorption of the bacteriophage to the cell, mechanisms that inhibit the injection of DNA into the cell, restriction-modification systems, and numerous systems that abort the infection at various points in the replication cycle [160,161,162]. Additionally, Hazan and Engelberg-Kulka [163] demonstrated that *E. coli mazEF*-mediated cell death acts as a suicide-defense mechanism to protect the bacterial culture against the spread of P1 phage infection.

## 5. Hurdle Technologies and Cross Protection

The adaptation of bacterial cells to a certain stress is often associated with enhanced protection against other subsequent stresses, which is referred to as “cross protection” [164]. This has important implications in food safety and risk assessment programs, given that preservative tools (stresses) applied for different food products are designed to eliminate microbial loads that have been grown under optimal rather than stress conditions [165]. Several stresses have been shown to induce cross protection, including heat stress [166], cold stress [167], acid stress [168] and osmotic stress [169].

Instead of one robust method such as heat sterilisation, minimal processing involves the use of a number of synergic mild preservation techniques known as hurdles. According to this approach we can look to cross protection as “hurdle stresses” to better understand the effectiveness and the challenges of hurdle technologies. While individually not effective in preventing microbial growth, the right combination of hurdles is a very powerful tool in preventing microbial outgrowth and in minimising organoleptic changes in foods.

The molecular basis of cross response and cross response genes are widely analysed in model Gram-negative and Gram-positive pathogenic bacteria. For instance, CspC and CspE from *E. coli* regulate the expression of RpoS-regulated stress proteins, such as OsmY, Dps, ProP and KatG, possibly thorough regulation of RpoS itself. These proteins are induced in response to osmotic stress, oxidative stress, or upon stationary phase. CspE and CspC also regulate expression of Universal protein A, UspA, a protein responding to numerous stresses [170]. In addition, CspA homologues are involved in diverse phenomena, such as response to freezing conditions, stationary phase, osmotic stress, starvation, antibiotic biosynthesis, resistance to antimicrobial peptides, inhibition of replication, heat resistance of the spores, UV sensitivity etc. [171,172,173,174]. *L. monocytogenes* possesses three small, highly homologous protein members of the cold shock protein (Csp) family. Schmid *et al.* [175] used gene expression analysis and a set of mutants with single, double, and triple deletions of the *csp* genes to evaluate the roles of CspA, CspB, and CspD in the cold and osmotic (NaCl) stress adaptation responses of *L. monocytogenes*. The hierarchies of their functional importance differed, depending on the environmental stress conditions: CspA>CspD>CspB in response to cold stress versus CspD>CspA/CspB in response to NaCl salt osmotic stress. The fact that Csps are promoting *L. monocytogenes* adaptation against both cold and NaCl stress has significant implications in view of practical food microbial control measures, in fact the combined or sequential exposure of *L. monocytogenes* cells to these two stresses in food environments might inadvertently induce cross-protection responses.

Among other factors, adaptation to heat stress appears to provide bacterial cells with more pronounced cross protection against several other stresses [176]. The ability of chaperones, both protein-based and chemical, to confer cold tolerance in bacteria might be maneuvered to improve the growth rate at low temperatures of some mesophilic bacteria [177]. Heat adaptation of *Salmonella* is reported to provide protection against subsequent heat treatment [178] and low pH conditions. Similarly, *S. typhimurium* showed increased resistance to heat and salt following adaptation to acidic condition [47]. This was linked to the observation that the expression of about half the acid shock proteins induced following exposure to acidic conditions were also stimulated by subjecting cells to heat shock [179]. Rowbury [64] reported that damage to DNA is probably the most lethal event in thermal inactivation. Single strand breaks were shown in heat-treated *S. typhimurium* and other microorganisms. Addition of NaCl (lowering a_w_) markedly protected DNA against loss of biological activity after heating at 121°C for 15 minutes [180]; probably, NaCl inactivated nuclease rather than lowering a_w_ per se. It is reported that the master stress regulator RpoS may be involved in mediating cross protection in bacteria. This is indicated by the increased level of the alternative sigma factor σs (encoded by the *rpoS* gene) following exposure to stresses such as osmotic stress, heat stress and starvation [64,108].

El-Sharoud [181] reported that increasing acid resistance of a given bacterium following exposure to other stressful conditions differs among bacteria species. Kim *et al.* [182] and Abram *et al.* [183] reported that sigma factor genes *sigh* (heat shock), *sigR* (oxidative stress), *sigB* (osmotic shock), and *hrdD*, which plays a major role in the secondary metabolism, were all strongly upregulated by the pH shock. A number of heat shock proteins including the DnaK family and chaperones such as GroEL were also observed to be upregulated by the pH shock, while their repressor *hspR* was strongly downregulated. Oxidative stress-related proteins such as thioredoxin, catalase, superoxide dismutase, peroxidase, and osmotic shock-related protein, such as vesicle synthases, were also upregulated in overall. An interlink between the cold tolerance and acid tolerance of *Lactobacillus delbrueckii* has been evidenced very recently by the enhanced freeze-tolerance of some cells that were acidified at pH 5.25 for 30 min at the end of fermentation [184].

Studying alternative sigma factor interactions in *Salmonella* during oxidative stress, Bang *et al.* [185] discovered that interactions between alternative sigma factors permitted the integration of diverse stress signals to produce coordinated genetic responses, suggesting the hierarchical interactions between alternative sigma factors control sequential gene expression in Gram-positive bacteria, whereas alternative sigma factors in Gram-negative bacteria are generally regarded to direct expression of discrete gene subsets. This consideration is confirmed in *Mycobacterium smegmatis*, where the alternative sigma factor SigF is required for survival to heat shock, acidic pH and oxidative stress [186]. The response of aerobically grown *E. coli* cells to the cold shock induced by the rapid lowering of growth temperature from 37 to 20 °C was found to be basically the same as the oxidative stress response [187].

Proteolysis is a powerful mechanism used by cells to control adaptation and recovery after exposure to a variety of stress conditions, first of all characteristic of heat stress response. *E. coli* has five ATP-dependent proteases: ClpAP, ClpXP, FtsH, HslUV and Lon [188,189,190]. A proteomic study indicated that UvrA is a substrate for degradation by ClpXP [191]. During post-UV recovery, UvrA levels decrease principally as a result of ClpXP-dependent protein degradation, revealing that a complex network of interactions contribute to tuning the level of UvrA in the cell in response to the extent of DNA damage [192].

With respect to solvent stress, among the three characterized small heat shock genes from *Lactobacillus plantarum* [193,194], Fiocco *et al*. [195] suggested a potential role for Hsp 18.55 and Hsp 19.3 (small Heat shock proteins) in solvent tolerance. In fact, overproduction of Hsp 18.55 and Hsp 19.3 led to an enhanced survival in the presence of butanol (1% v/v) or ethanol (12% v/v) treatment.

Concerning high pressure and cross-protection, under high hydrostatic pressure the syntheses of some HSPs and CSPs were found to be induced in *E. coli* [196]. Bacterial ribosomes seem to play the role of intracellular sensors, which integrate the adaptation of the organism to high temperatures, low temperatures and high pressure. In contrast, differences in pressure tolerance of *L. monocytogenes* strains are not correlated with other stress tolerances [197].

McDougald *et al.* [198] have found evidence for a large degree of overlap in the cell’s use of global regulators to deal with both starvation and oxidative stress. In addition, the post-transcriptional regulator CsrA (or RsmA) has been reported to play a central role in cross protection (starvation, oxidative stress, virulence) and in the adaptation of baterial pathogens to different stages of infection in animals and also in vegetable/fruit [199].

Giotis *et al.* [200], in *L. monocytogenes*, found that alkaline conditions induced cross-protection against osmotic and ethanol challenges; this phenomenon may have serious implications for food safety and human health because such stress conditions are routinely used as part of food preservation and surface cleaning processes.

With respect to biological compounds, we may only remember that: i) it is also well documented that some compounds called chemical chaperones (e.g. glycine, betaine and proline), which are known to stabilize the native conformation of cellular proteins, were found to have a protective role against cold stress, salt stress and thermal stress in bacteria [201,202,203]; ii) treating bacterial cells with two different groups of antibiotics (which all acted on ribosomes), which were found to mimic temperature upshift and downshift of *E. coli* cells, led to the synthesis of HSPs and cold shock proteins (CSPs), respectively [204].

With regards to biotic stressor, we only underline that *psp* operon induction, first depicted as a response of *E. coli* upon infection with filamentous phages [205], was oserved also under more general stress conditions, including extreme heat shock, hyperosmotic stress, ethanol treatment, and uncoupling of proton motive force [206,207].

## 6. Conclusions

Minimally processed food is easily contaminated by food borne pathogens either directly or via cross-contamination during food preparation. For instance, in addition to *Salmonella*, *L. monocytogenes* and *E.coli* O157:H7, fresh cut produce have been identified as a transmission vehicle for pathogens such as *Campylobacter* species [208]. The ability of pathogens to survive stress requires specific, co-ordinated responses, which induce resistance to the stressful conditions. The molecular mechanisms involved are complex and there are a number of genes involved in bacterial stress response. For instance, the ability of *L. monocytogenes* and several Gram-positive bacteria (such as *B. subtilis* and *Staphylococcus aureus*) to resist many adverse environmental conditions has been attributed in part to activation of the alternative sigma factor σ^B^, encoded by the *sigB* gene. Survival under stress involves adaptive responses mediated by a set of conserved proteins (usualy called heat-shock proteins), that are upregulated upon exposure to heat shock, low pH, oxidative agents, toxic chemical compounds, starvation, and in general, any situation in which bacterial growth is arrested indicating a protective role in the general stress response.

Apart from the enhanced survival in foods and increased resistance to subsequent food processing treatments, adapted or hardened pathogens may also have enhanced virulence. Stress response and cross-protection must be considered when current processing technologies are being modified or when new preservation technologies are being developed for fresh-cut produce. These responses are particularly significant in minimal processing technologies used in preparation of fresh-cut produce, where the imposition of one sub-lethal stress may lead to the induction of multiple stress responses that may reduce the efficacy of subsequent treatments [209,210,211]. More research on how to use cumulative sub-lethal hurdles and safe practical interventions, without inducing stress response, is needed.

Finally, the combination of well designed integrated production, handling, processing and distribution chains for fresh-cut produces is crucial for achieving the high quality and safety demanded by consumers [212]. One important strategy might be studying the molecular basis of cross response of human pathogens to develop the most suitable combination of synergic “hurdles”. Moreover, we should take into account that synergic and antagonistic actions of hurdle technologies may be pathogen dependent, and that selected hurdle technology combinations may also improve the bacterial pathogens ability to survive gastric acid conditions without enhancing virulence.
